# Supplementary material for: Estimated glucose disposal rate and risk of metabolic syndrome: A population-based study
Source: PLoS One. 2025 Oct 31;20(10):e0335502. doi: 10.1371/journal.pone.0335502 (PMC12578165; doi:10.1371/journal.pone.0335502)
Supplement: S1 File — (DOCX) [file pone.0335502.s001.docx]

Table A.1 Baseline characteristic of the study population according to eGDR.

|  | Total  (n=3229) | 1  (n = 808) | 2  (n = 807) | 3  (n = 807) | 4  (n = 807) | *P* |
| --- | --- | --- | --- | --- | --- | --- |
| Age, years | 58 (52, 65) | 60 (53, 66) | 57 (51, 63) | 58 (53, 65) | 57 (51, 64) | < 0.001 |
| Sex, n (%) |  |  |  |  |  | < 0.001 |
| Female | 1518 (47.01) | 390 (48.27) | 354 (43.87) | 350 (43.37) | 424 (52.54) |  |
| Male | 1711 (52.99) | 418 (51.73) | 453 (56.13) | 457 (56.63) | 383 (47.46) |  |
| Marital status, n (%) |  |  |  |  |  | < 0.001 |
| Non-Married | 460 (14.25) | 128 (15.84) | 83 (10.29) | 108 (13.38) | 141 (17.47) |  |
| Married | 2769 (85.75) | 680 (84.16) | 724 (89.71) | 699 (86.62) | 666 (82.53) |  |
| Location, n (%) |  |  |  |  |  | 0.032 |
| City/Town | 891 (27.59) | 254 (31.44) | 208 (25.77) | 222 (27.51) | 207 (25.65) |  |
| Village | 2338 (72.41) | 554 (68.56) | 599 (74.23) | 585 (72.49) | 600 (74.35) |  |
| Education, n (%) |  |  |  |  |  | 0.253 |
| Below primary school | 1538 (47.68) | 396 (49.07) | 354 (43.98) | 377 (46.72) | 411 (50.93) |  |
| Primary school | 757 (23.47) | 175 (21.69) | 207 (25.71) | 199 (24.66) | 176 (21.81) |  |
| Middle school | 653 (20.24) | 167 (20.69) | 176 (21.86) | 161 (19.95) | 149 (18.46) |  |
| High school and above | 278(8.62) | 69(8.55) | 68(8.45) | 70(8.67) | 71 (8.80) |  |
| Drink status, n (%) |  |  |  |  |  | 0.056 |
| Never | 1777 (55.07) | 436 (54.03) | 446 (55.27) | 428 (53.04) | 467 (57.94) |  |
| Former | 243 (7.53) | 79 (9.79) | 55 (6.82) | 61 (7.56) | 48 (5.96) |  |
| Current | 1207 (37.40) | 292 (36.18) | 306 (37.92) | 318 (39.41) | 291 (36.10) |  |
| Smoke status, n (%) |  |  |  |  |  | 0.001 |
| Never | 1803 (55.99) | 456 (56.65) | 456 (56.58) | 424 (52.67) | 467 (58.08) |  |
| Former | 250 (7.76) | 85 (10.56) | 62 (7.69) | 60 (7.45) | 43 (5.35) |  |
| Current | 1167 (36.24) | 264 (32.8) | 288 (35.73) | 321 (39.88) | 294 (36.57) |  |
| Sleep time, hours | 6 (5, 8) | 6 (5, 8) | 7 (5, 8) | 6 (5, 8) | 6 (5, 8) | 0.493 |
| Hypertension, n (%) |  |  |  |  |  | < 0.001 |
| No | 2871 (88.91) | 458 (56.68) | 807 (100.00) | 807 (100.00) | 799 (99.01) |  |
| Yes | 358 (11.09) | 350 (43.32) | 0 (0.00) | 0 (0.00) | 8 (0.99) |  |
| TG (mg/dL) | 84.96  (65.27, 112.39) | 89.39  (68.14, 113.28) | 86.73  (65.49, 112.39) | 84.07  (64.61, 111.51) | 80.54  (62.83, 109.74) | 0.003 |
| HbA1C (%) | 5.1 (4.8, 5.3) | 5.2 (4.9, 5.4) | 5.1 (4.9, 5.4) | 5.1 (4.9, 5.3) | 4.9 (4.7, 5.2) | < 0.001 |
| CRP (mg/L) | 0.77 (0.46,1.55) | 0.91 (0.54, 1.78) | 0.75 (0.47, 1.51) | 0.73 (0.45, 1.46) | 0.68 (0.41, 1.37) | < 0.001 |
| BMI (kg/m2) | 21.66  (19.99, 23.67) | 23.98  (21.52, 26.31) | 22.72  (21.39, 24.01) | 21.1  (19.98, 22.25) | 19.76  (18.43, 21.2) | < 0.001 |
| WC (cm) | 79.8  (75.00, 85.00) | 89.45  (80.17, 93.80) | 84  (82.00, 86.20) | 78  (76.20, 80.00) | 72  (69.40, 74.00) | < 0.001 |
| eGDR (mg/kg/min) | 11.05  (10.35, 11.62) | 9.32  (7.90, 10.01) | 10.76  (10.58, 10.91) | 11.33  (11.19, 11.47) | 11.96  (11.77, 12.27) | < 0.001 |
| MetS, n (%) |  |  |  |  |  | < 0.001 |
| No | 2484 (76.93) | 447 (55.32) | 621 (76.95) | 694 (86.00) | 722 (89.47) |  |
| Yes | 745 (23.07) | 361 (44.68) | 186 (23.05) | 113 (14.00) | 85 (10.53) |  |

Variables are presented as median (interquartile range, IQR). P-values were calculated from chi-square tests (categorical variables) or rank-sum tests (continuous variables without normal distribution), or t-tests (continuous variables with normal distribution).

TG, Triglyceride; HbA1C, Glycated Hemoglobin A1C; CRP, C-Reactive Protein; WC, Waist Circumference; eGDR, Estimated Glucose Disposal Rate; MetS, Metabolic Syndrome.

Table A.2 Mediation of the association between eGDR and MetS by BMI

|  | Estimate | 95% CI Lower | 95% CI Upper | P-value |
| --- | --- | --- | --- | --- |
| ACME (average) | -0.00252 | -0.00662 | 0.00000 | <0.001 |
| ADE (average) | -0.00547 | -0.00991 | 0.00000 | <0.001 |
| Prop. Mediated | 0.29077 | 0.19421 | 0.42000 | <0.001 |

CI, confidence interval; eGDR, Estimated Glucose Disposal Rate; MetS, Metabolic Syndrome; ADE, Average Direct Effect; ACME, Average Causal Mediation Effect.

Table A.3 Predictive values of eGDR, WC, and HbA1C for MetS

|  | AUC (95%CI) | Accuracy (95%CI) | Sensitivity (95%CI) | Specificity (95%CI) | PPV (95%CI) | NPV (95%CI) | Cut off |
| --- | --- | --- | --- | --- | --- | --- | --- |
| eGDR | 0.71 (0.69-0.73) | 0.31 (0.29-0.32) | 0.28  (0.26 -0.30) | 0.39  (0.36 - 0.43) | 0.61  (0.58 - 0.63) | 0.14  (0.13 - 0.16) | 10.70 |
| WC | 0.67 (0.65-0.69) | 0.67 (0.65-0.68) | 0.69  (0.68 - 0.71) | 0.57  (0.54 - 0.61) | 0.84  (0.83 - 0.86) | 0.36  (0.33 - 0.39) | 82.65 |
| HbA1C | 0.52 (0.49-0.54) | 0.61 (0.59-0.63) | 0.69  (0.67 - 0.71) | 0.34  (0.31 - 0.38) | 0.78  (0.76 - 0.80) | 0.25  (0.22 - 0.28) | 5.25 |
