# Supplementary figures and images for: Estimated glucose disposal rate and risk of metabolic syndrome: A population-based study
Source: PLoS One. 2025 Oct 31;20(10):e0335502. doi: 10.1371/journal.pone.0335502 (PMC12578165; doi:10.1371/journal.pone.0335502)

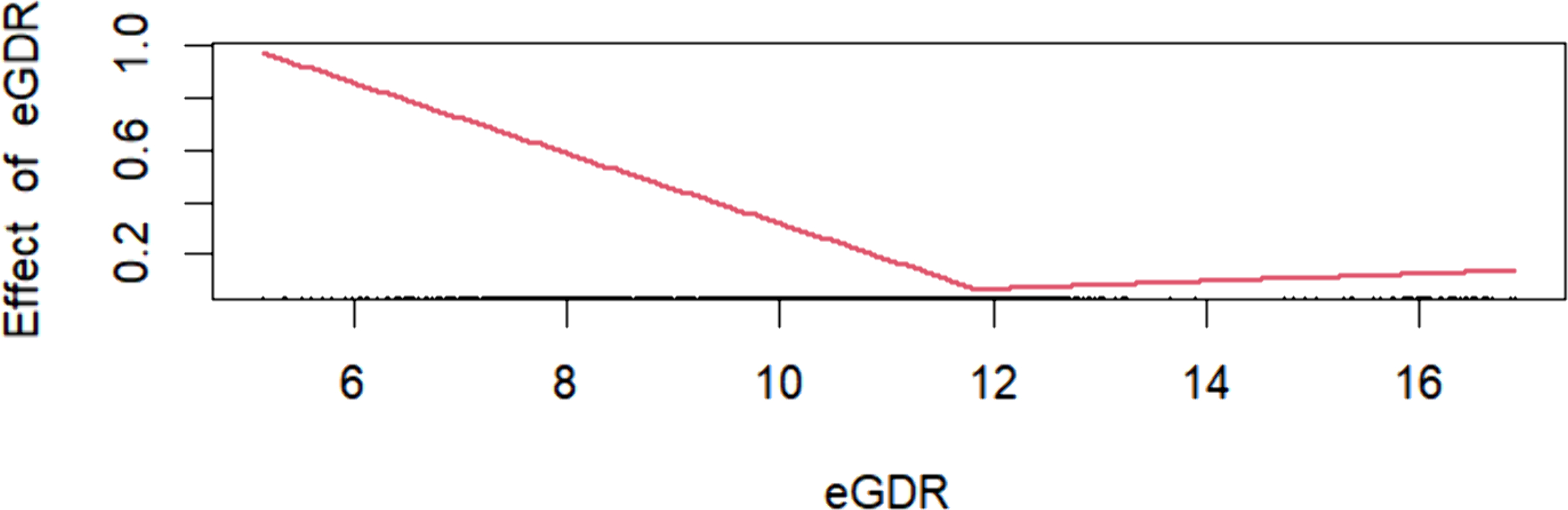

Supplement: S1 Fig — (TIF) [file pone.0335502.s002.tif]
