# Supplementary material for: Estimated glucose disposal rate and risk of metabolic syndrome: A population-based study
Source: PLoS One. 2025 Oct 31;20(10):e0335502. doi: 10.1371/journal.pone.0335502 (PMC12578165; doi:10.1371/journal.pone.0335502)
Supplement: Attachment — Submitted filename: Zhang et al., Higher Estimated Glucose Disposal Rate.pdf [file pone.0335502.s003.pdf]

PLOS ONE

Higher Estimated Glucose Disposal Rate Is Associated with Reduced Risk of Metabolic Syndrome: A Population-Based Study

--Manuscript Draft--

**Manuscript Number:**	PONE-D-25-26075
**Article Type:**	Research Article
**Full Title:**	Higher Estimated Glucose Disposal Rate Is Associated with Reduced Risk of Metabolic Syndrome: A Population-Based Study
**Short Title:**	Higher Estimated Glucose Disposal Rate Is Associated with Reduced Risk of Metabolic Syndrome
**Corresponding Author:**	Bohai Yu<br>Shenzhen Hospital(Futian) of Guangzhou University of Chinese Medicine (Futian)<br>Shenzhen, CHINA
**Keywords:**	CHARLS, Estimated Glucose Disposal Rate, Metabolic Syndrome, Longitudinal Study, Mediation Analysis
**Abstract:**	Aims: This study aims to explore the association between the estimated glucose disposal rate (eGDR) and the risk of metabolic syndrome (MetS), with a focus on the mediating role of BMI.<br>Methods: Data for this study came from the 2011 and 2015 China Health and Retirement Longitudinal Study. We used multivariable logistic regression and restricted cubic splines to assess the relationship between eGDR and MetS, with subgroup and interaction analyses to identify moderating factors. The diagnostic ability of eGDR for MetS was evaluated via receiver operating characteristic curves.<br>Results: In total, 3,229 participants were included, with 745 (23.07%) diagnosed with MetS. In the fully adjusted model, each interquartile range increase in eGDR was associated with a 58% reduced MetS risk (odds ratio = 0.42, 95% confidence interval: 0.36–0.49, P < 0.001). A significant nonlinear dose–response relationship between eGDR and MetS risk was observed (P < 0.001, both overall and nonlinear).Spline regression analysis further revealed that the protective effect of eGDR was significant up to 11.88 mg/kg/min (standard error = 0.17). Subgroup analysis revealed significant interaction effects of marital status and residential area on the eGDR–MetS relationship (P < 0.05), while BMI mediated 29% of eGDR's total effect on MetS. Receiver operating characteristic analysis showed eGDR's good predictive performance for MetS, with an area under the curve of 0.71 (95% confidence interval: 0.69–0.73).<br>Conclusion:Higher eGDR levels were linked to a significantly lower MetS risk, suggesting individuals with low eGDR should monitor their potential for MetS development.
**Order of Authors:**	Lin Zhang
	Nian Cai
	Li Mo
	Xiaofang Tian
	Bohai Yu
**Additional Information:**	
**Question**	**Response**
**Financial Disclosure**<br><br>Enter a financial disclosure statement that describes the sources of funding for the work included in this submission. Review the submission guidelines for detailed requirements. View published research	The author(s) received no specific funding for this work.

articles from *PLOS ONE* for specific examples.<br><br>This statement is required for submission and **will appear in the published article** if the submission is accepted. Please make sure it is accurate.<br><br>**Funded studies**<br>Enter a statement with the following details:<br>• Initials of the authors who received each award<br>• Grant numbers awarded to each author<br>• The full name of each funder<br>• URL of each funder website<br>• Did the sponsors or funders play any role in the study design, data collection and analysis, decision to publish, or preparation of the manuscript?<br><br>Did you receive funding for this work?	
**Competing Interests**<br><br>Use the instructions below to enter a competing interest statement for this submission. On behalf of all authors, disclose any competing interests that could be perceived to bias this work—acknowledging all financial support and any other relevant financial or non-financial competing interests.<br><br>This statement is required for submission and **will appear in the published article** if the submission is accepted. Please make sure it is accurate and that any funding sources listed in your Funding Information later in the submission form are also declared in your Financial Disclosure statement.<br><br>View published research articles from *PLOS ONE* for specific examples.	The authors have declared that no competing interests exist.

**NO authors have competing interests**<br><br>Enter: *The authors have declared that no competing interests exist.*<br><br>**Authors with competing interests**<br><br>Enter competing interest details beginning with this statement:<br><br>*I have read the journal's policy and the authors of this manuscript have the following competing interests: [insert competing interests here]*<br><br>* typeset	
**Ethics Statement**<br><br>Enter an ethics statement for this submission. This statement is required if the study involved:<br><br>• Human participants<br>• Human specimens or tissue<br>• Vertebrate animals or cephalopods<br>• Vertebrate embryos or tissues<br>• Field research<br><br>Write "N/A" if the submission does not require an ethics statement.<br><br>General guidance is provided below. Consult the submission guidelines for detailed instructions. **Make sure that all information entered here is included in the Methods section of the manuscript.**	NA

**Format for specific study types**<br><br>**Human Subject Research (involving human participants and/or tissue)**<br>• Give the name of the institutional review board or ethics committee that approved the study<br>• Include the approval number and/or a statement indicating approval of this research<br>• Indicate the form of consent obtained (written/oral) or the reason that consent was not obtained (e.g. the data were analyzed anonymously)<br><br>**Animal Research (involving vertebrate animals, embryos or tissues)**<br>• Provide the name of the Institutional Animal Care and Use Committee (IACUC) or other relevant ethics board that reviewed the study protocol, and indicate whether they approved this research or granted a formal waiver of ethical approval<br>• Include an approval number if one was obtained<br>• If the study involved *non-human primates*, add *additional details* about animal welfare and steps taken to ameliorate suffering<br>• If anesthesia, euthanasia, or any kind of animal sacrifice is part of the study, include briefly which substances and/or methods were applied<br><br>**Field Research**<br><br>Include the following details if this study involves the collection of plant, animal, or other materials from a natural setting:<br>• Field permit number<br>• Name of the institution or relevant body that granted permission	
**Data Availability**<br><br>Authors are required to make all data underlying the findings described fully available, without restriction, and from the time of publication. PLOS allows rare exceptions to address legal and ethical concerns. See the PLOS Data Policy and FAQ for detailed information.	Yes - all data are fully available without restriction

A Data Availability Statement describing where the data can be found is required at submission. Your answers to this question constitute the Data Availability Statement and **will be published in the article**, if accepted.<br><br>**Important:** Stating 'data available on request from the author' is not sufficient. If your data are only available upon request, select 'No' for the first question and explain your exceptional situation in the text box.<br><br>Do the authors confirm that all data underlying the findings described in their manuscript are fully available without restriction?	
**Describe where the data may be found in full sentences. If you are copying our sample text, replace any instances of XXX with the appropriate details.**<br><br>• If the data are **held or will be held in a public repository**, include URLs, accession numbers or DOIs. If this information will only be available after acceptance, indicate this by ticking the box below. For example: *All XXX files are available from the XXX database (accession number(s) XXX, XXX.).*<br>• If the data are all contained **within the manuscript and/or Supporting Information files**, enter the following: *All relevant data are within the manuscript and its Supporting Information files.*<br>• If neither of these applies but you are able to provide **details of access elsewhere**, with or without limitations, please do so. For example:<br><br>*Data cannot be shared publicly because of [XXX]. Data are available from the XXX Institutional Data Access / Ethics Committee (contact via XXX) for researchers who meet the criteria for access to confidential data.*<br><br>*The data underlying the results presented in the study are available from (include the name of the third party*	The datasets supporting the conclusions of this article are available publicly, http://charls.pku.edu.cn/pages/data/111/en.html.

Powered by Editorial Manager® and ProduXion Manager® from Aries Systems Corporation

and contact information or URL).<br>• This text is appropriate if the data are owned by a third party and authors do not have permission to share the data.<br><br>* typeset	
Additional data availability information:	Tick here if your circumstances are not covered by the questions above and you need the journal’s help to make your data available.

Powered by Editorial Manager® and ProduXion Manager® from Aries Systems Corporation

Manuscript Click here to access/download;Manuscript;Manuscript.docx

Higher Estimated Glucose Disposal Rate Is Associated with Reduced Risk of Metabolic Syndrome: A Population-Based Study

Lin Zhang[1], Nian Cai[1], Li Mo[1], Xiaofang Tian[1], Bohai Yu[1]*

1 Shenzhen Hospital (Futian) of Guangzhou University of Chinese Medicine, NO. 6001 of Beihuan Road, Shenzhen, Guangdong, 518000, China

Lin Zhang: lynnzhang@163.com
Nian Cai: niancaiCN@outlook.com
Li Mo: 1936262213@qq.com
Xiaofang Tian: 1329420611@qq.com
*Corresponding Author Bohai Yu: wwwcnwxx6688@163.com
Permanent address: Shenzhen Hospital (Futian) of Guangzhou University of Chinese Medicine, NO. 6001 of Beihuan Road, Shenzhen, Guangdong, 518000, China

Aims: This study aims to explore the association between the estimated glucose disposal rate (eGDR) and the risk of metabolic syndrome (MetS), with a focus on the mediating role of BMI.
Methods: Data for this study came from the 2011 and 2015 China Health and Retirement Longitudinal Study. We used multivariable logistic regression and restricted cubic splines to assess the relationship between eGDR and MetS, with subgroup and interaction analyses to identify moderating factors. The diagnostic ability of eGDR for MetS was evaluated via receiver operating characteristic curves.
Results: In total, 3,229 participants were included, with 745 (23.07%) diagnosed with MetS. In the fully adjusted model, each interquartile range increase in eGDR was associated with a 58% reduced MetS risk (odds ratio = 0.42, 95% confidence interval: 0.36–0.49, P < 0.001). A significant nonlinear dose–response relationship between eGDR and MetS risk was observed (P < 0.001, both overall and nonlinear).Spline regression analysis further revealed that the protective effect of eGDR was significant up to 11.88 mg/kg/min (standard error = 0.17). Subgroup analysis revealed significant interaction effects of marital status and residential area on the eGDR–MetS relationship (P < 0.05), while BMI mediated 29% of eGDR's total effect on MetS. Receiver operating characteristic analysis showed eGDR's good predictive performance for MetS, with an area under the curve of 0.71 (95% confidence interval: 0.69–0.73).
Conclusion: Higher eGDR levels were linked to a significantly lower MetS risk, suggesting individuals with low eGDR should monitor their potential for MetS development.
Keywords: CHARLS, Estimated Glucose Disposal Rate, Metabolic Syndrome,

Longitudinal Study, Mediation Analysis

Introduction

Metabolic syndrome (MetS), also referred to as Syndrome X or insulin resistance syndrome, represents a cluster of cardiovascular risk factors rather than a single disease.(1) The pathophysiology of MetS is intricate and not yet fully elucidated, and its diagnostic criteria have undergone continuous refinement since the 1980s.(2)Although several organizations have proposed varying diagnostic criteria, the differences between criteria are generally minor. Despite the limited availability of global data on MetS, its prevalence is estimated to be approximately three times that of diabetes, suggesting that around one-quarter of the global population—more than one billion individuals—is affected.(3) This syndrome not only presents significant health risks but also imposes a massive economic burden, amounting to trillions of dollars in healthcare costs and potential economic productivity losses.(4)

To accurately assess the risk of MetS, researchers have developed various evaluation metrics.(5, 6) Among these, the estimated glucose disposal rate (eGDR), which incorporates waist circumference (WC), hypertension, and glycated hemoglobin (HbA1c), is used to assess individual insulin sensitivity.(7) As a continuous, simple, and non-invasive measure, eGDR has been widely adopted in clinical research. Previous studies have demonstrated that lower eGDR values are associated with significantly increased risks of renal disease, peripheral vascular disease, coronary artery disease, and mortality.(8-11) However, the specific relationship between eGDR and MetS has not yet been fully established.

Therefore, this study aims to investigate the role of dynamic changes in eGDR in predicting MetS through a prospective cohort study. By systematically evaluating the impact of eGDR variations on the risk of MetS, the study seeks to provide a scientific basis for the early identification and intervention of MetS.

Method

Study Population

The China Health and Retirement Longitudinal Study(CHARLS) is a survey targeting middle-aged and older adults in China, specifically, individuals aged 45 years and above.(12) The survey aims to establish a high-quality micro-database that encompasses a wide range of socioeconomic and health-related information to support research on aging. The national baseline survey of China Health and Retirement Longitudinal Study was conducted between 2011 and 2012, covering 28 provinces, 150 counties/districts, and 450 villages/communities. A total of 17,708 individuals from 10,257 households were interviewed. Institutionalized elderly individuals were not included in the initial sampling; however, baseline respondents who later moved into institutions were followed up in subsequent surveys. The second wave of data collection took place in 2013, with no biomarkers collected during this follow-up period. The third wave, primarily conducted between July and October 2015, included the collection of blood samples as in the first wave. The fourth wave was conducted in 2018. The Ethics Review Committee of Peking University approved the study protocol, and all participants provided written informed consent.

The study population comprised participants from the first and third waves of the

survey who had available blood test data.(13) Participants were required to fast overnight before the blood draw. The blood samples were collected by nurses from local community hospitals or clinics hired by the research team. Three tubes of venous blood were collected from each participant, and after preliminary processing, the samples were transported directly to Peking University via a cold chain for further analysis.

In accordance with the study objectives, participants with incomplete baseline eGDR data (n=7,844), a baseline diagnosis of MetS (n=4,750), use of glucose-lowering medications (n=71), age below 45 years or missing age information (n=90), and incomplete MetS outcome data were excluded (n=1,724). This left a final set of 3,229 participants for analysis (Fig. 1).

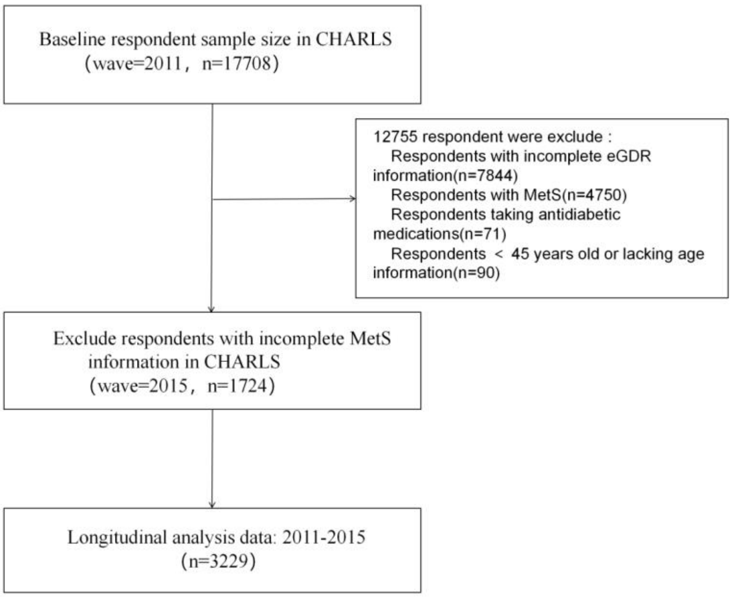

Fig.1 Flowchart illustrating the respondents selection process.

Measurement

In this study, the eGDR was calculated using a previously described formula:(14) eGDR (mg/kg/min) = 21.158 − (0.09 × WC) − (3.407 × hypertension) − (0.551 × HbA1c), where WC was measured in cm, hypertension was coded 1 for yes and 2 for no, and HbA1c was included as a percentage.

Outcome

The diagnosis of MetS was based on criteria defined by Chinese standards,(15)which include five components:

(1) Central obesity: WC ≥ 80 cm for women and ≥ 90 cm for men;

(2) Elevated triglyceride (TG) levels: TG levels ≥ 150 mg/dL;

(3) Low high-density lipoprotein cholesterol levels: high-density lipoprotein cholesterol levels < 40 mg/dL for men and < 50 mg/dL for women;

(4) Elevated blood pressure (BP): systolic BP ≥ 130 mmHg and/or diastolic BP ≥ 85 mmHg, or use of antihypertensive treatment;

(5) Elevated fasting plasma glucose (FPG): FPG levels ≥ 100 mg/dL, use of antidiabetic

medication, or self-reported diabetes history.

A diagnosis of MetS is made if at least three of these five criteria are present.

Covariate Assessment

We reviewed baseline demographic information, including age, sex, residence location (rural/urban), education level (below primary school/primary school/junior high school/high school and above), marital status (married/unmarried), and sleep duration. Basic anthropometric measurements such as height, weight, and WC were collected, along with potential risk factors including smoking status (never smoked/former smoker/current smoker) and alcohol consumption status (never drank/former drinker/current drinker). Laboratory tests included FPG, TG, C-reactive protein (CRP), and HbA1c. Additionally, self-reported data on hypertension history and use of antidiabetic medications were collected.

Statistical Analyses

Baseline characteristics of non-normally distributed data were analyzed using the median and interquartile range (Q1–Q3). Categorical variables are presented as percentages. To compare data based on eGDR quartiles and the presence of MetS, we used t-tests, Mann–Whitney U tests, or chi-square tests as appropriate. Three logistic regression models were employed to calculate the odds ratios (OR) and 95% confidence intervals (CIs) for MetS, analyzing eGDR both as a continuous variable (per interquartile range increase) and as a categorical variable (quartiles). In Model 1, we assessed the crude association between eGDR and MetS. Model 2 included adjustments for age, sex, marital status, education level, residence location, sleep duration, smoking, and alcohol consumption. Model 3 included further adjustments for TG, CRP, HbA1c, and hypertension.

This study explored the dose–response relationship between eGDR and MetS, visualizing it through spline regression analysis to identify critical points and investigate the nonlinear relationship. Additionally, we conducted subgroup and interaction analyses based on age, sex, marital status, education level, residence location, smoking, alcohol consumption, and sleep duration to comprehensively assess the association between eGDR and MetS.

Additionally, we performed mediation analysis using the R package "mediation" to examine the mediating role of BMI in the relationship between eGDR and MetS. To further evaluate the diagnostic performance of eGDR in predicting MetS, we conducted receiver operating characteristic (ROC) curve analysis to determine its accuracy in diagnosing MetS.

All statistical analyses were performed using R software version 4.1, with a two-tailed *P*-value < 0.05 considered statistically significant.

Results

Characteristics of the Study Population According to MetS Status

The study included a total of 3,229 participants with a mean age of 58 years. Among them, 1,711 (53.0%) were male, and 1,518 (47.0%) were female. During the long-term follow-up from 2011 to 2015, 745 participants (23.0%) developed MetS.

Table 1 presents the baseline characteristics of the participants, categorized by the presence or absence of MetS. Participants with MetS had significantly higher levels of

TG and CRP and a significantly higher BMI and WC than those without MetS ($P < 0.001$). The prevalence of MetS was also significantly higher among female participants (57.58% vs. 42.42%, $P < 0.001$). There were statistically significant differences between the MetS and Non-MetS groups in terms of smoking (63.02% vs. 53.98%, $P < 0.001$), drinking (60.22% vs. 53.52%, $P < 0.001$), and hypertension (75.70% vs. 24.30%, $P < 0.001$). Notably, we observed that individuals with MetS had significantly lower eGDR levels compared with those without MetS (10.43 vs. 11.19, $P < 0.001$).

Table 1 Baseline characteristics of study population by MetS status at follow-up.

	Total (n = 3229)	Non-MetS (n = 2484)	MetS (n = 745)	*P*
Age	58 (52, 65)	58 (51.75, 64)	58 (52, 65)	0.283
Sex				< 0.001
Female	1518 (47.01)	1089 (43.84)	429 (57.58)	
Male	1711 (52.99)	1395 (56.16)	316 (42.42)	
Marital				0.066
Non-Married	460 (14.25)	338 (13.61)	122 (16.38)	
Married	2769 (85.75)	2146 (86.39)	623 (83.62)	
Location				0.114
City/Town	891 (27.59)	668 (26.89)	223 (29.93)	
Village	2338 (72.41)	1816 (73.11)	522 (70.07)	
Education				0.564
Below primary school	1538 (47.68)	1167 (47)	371 (49.93)	
Primary school	757 (23.47)	588 (23.68)	169 (22.75)	
Middle school	653 (20.24)	511 (20.58)	142 (19.11)	
High school and above	278 (8.62)	217 (8.74)	61 (8.21)	
Drink				< 0.001
Never drank	1777 (55.07)	1329 (53.52)	448 (60.22)	
Ever drank	243 (7.53)	182 (7.33)	61 (8.20)	
Current drinker	1207 (37.40)	972 (39.15)	235 (31.59)	
Smoke				< 0.001
Never smoked	1803 (55.99)	1336 (53.89)	467 (63.02)	
Ever smoked	250 (7.76)	197 (7.95)	53 (7.15)	
Current smoker	1167 (36.24)	946 (38.16)	221 (29.82)	
Sleep time	6 (5, 8)	6 (5, 8)	6 (5, 8)	0.795
Hypertion				< 0.001
No	2871 (88.91)	2307 (92.87)	564 (75.70)	
Yes	358 (11.09)	177 (7.13)	181 (24.30)	
TG (mg/dl)	84.96 (65.27,112.39)	81.42 (63.72,107.97)	98.24 (73.46,124.79)	< 0.001
HbA1C (%)	5.1 (4.8, 5.3)	5.1 (4.8, 5.3)	5.1 (4.9, 5.3)	0.192
CRP (mg/l)	0.77 (0.46, 1.55)	0.74 (0.45, 1.48)	0.89 (0.53, 1.78)	< 0.001
BMI（kg/m^2）	21.66 (19.99, 23.67)	21.23 (19.68, 23.09)	23.09 (21.46, 25.23)	< 0.001
WC（cm）	79.8 (75, 85)	79 (74, 84)	84 (78, 90)	< 0.001
eGDR (mg/kg/min)	11.05 (10.35,11.62)	11.19 (10.60, 11.70)	10.43 (8.93, 11.09)	< 0.001

p-Values were calculated from chi-square tests (categorical variables) or rank-sum tests (continuous variables without normal distribution), or t-tests (continuous variables with normaldistribution). TG, Triglyceride; HbA1C, Glycated Hemoglobin A1C; CRP, C-Reactive Protein; WC, Waist Circumference; eGDR, Estimated Glucose Disposal Rate; MetS, Metabolic Syndrome.

Characteristics of Study Population by eGDR Quartiles

Furthermore, we conducted an analysis of the population across different eGDR quartiles (Table A.1). Notably, as eGDR levels increased, there was a corresponding decline in the incidence of newly diagnosed MetS, with rates of 44.68%, 23.05%, 14.00%, and 10.53% across increasing quartiles (P < 0.001). Additionally, significant differences were observed among participants across various baseline characteristics, including age, gender, marital status, residence location, hypertension status, smoking, TG, CRP, HbA1c, BMI, and WC (all P < 0.05).

Associations Between Baseline eGDR and MetS Incidence

Table 3 outlines the association between eGDR levels and the risk of developing MetS. There was a significant inverse relationship between eGDR and the risk of MetS. Specifically, in the unadjusted model, compared with the lowest quartile of eGDR (Q1), participants in the second (Q2), third (Q3), and fourth (Q4) quartiles exhibited a progressively lower risk of MetS, with ORs and 95% CIs of 0.37 (95% CI: 0.30–0.46), 0.20 (95% CI: 0.16–0.26), and 0.15 (95% CI: 0.11–0.19), respectively. In Models 2 and 3, after adjusting for covariates, the OR for the Q3 group was no longer significantly lower than for the reference group (OR = 1.01 [95% CI: 1.00–1.02] in both models). In the fully adjusted model (Model 3), compared with the Q1 group, the Q2 and Q4 groups had a significantly lower risk of MetS (OR = 0.50 [95% CI: 0.45–0.57] and OR = 0.44 [95% CI: 0.33–0.59], respectively), with the trend analysis showing a statistically significant effect (P for trend < 0.001).

Figure 2 illustrates the dose–response relationship between eGDR and the risk of developing MetS. There was a significant nonlinear association between eGDR levels and the risk of MetS (P < 0.001 [overall], P < 0.001 [nonlinear]).According to the spline regression analysis, there was a strong negative association between eGDR and MetS up to an eGDR of 11.88 (standard error = 0.17). Beyond this point, the association weakened, with little to no additional protective effect observed (Fig. A.1).

Table 3 Associations between baseline eGDR with follow-up incident MetS

	Model 1	*P*	Model 2	*P*	Model 3	*P*
eGDR per IQR	0.53 (0.49,0.58)	<0.001	0.52 (0.49,0.57)	<0.001	0.42 (0.36,0.49)	<0.001
Q1	Ref		Ref		Ref	
Q2	0.37 (0.30,0.46)	<0.001	0.60 (0.57, 0.64)	<0.001	0.50 (0.45,0.57)	<0.001
Q3	0.20(0.16, 0.26)	<0.001	1.01 (1.00, 1.02)	0.161	1.01 (1.00,1.02)	0.03
Q4	0.15 (0.11, 0.19)	<0.001	0.45 (0.34, 0.60)	<0.001	0.44 (0.33, 0.59)	<0.001
P for trend	<0.001		<0.001		<0.001	

Model 1 was the unadjusted model. Model 2 was adjusted for age, gender, education level, location, marital status, smoking status, drinking status, and sleep time. Model 3 was further adjusted for hypertension, TG, CRP, and HbA1C based on Model 2.

eGDR, Estimated Glucose Disposal Rate; MetS, Metabolic Syndrome; IQR, Interquartile Range.

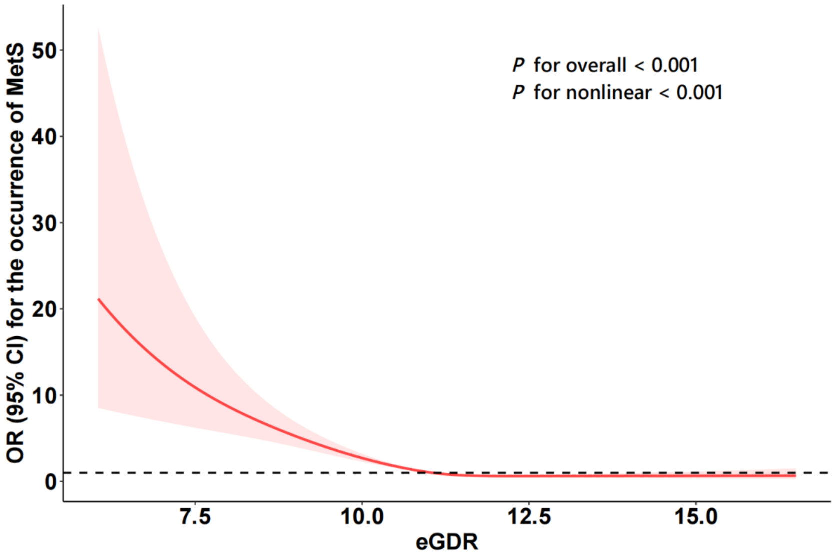

Fig. 2 Dose-Response Relationship Between eGDR and MetS.

Subgroup Analysis

To explore whether the association between eGDR and the incidence of MetS varies across different subgroups, we conducted analyses stratified by participants' socioeconomic characteristics and medical history (Fig. 3). There were significant interactions between eGDR and marital status and residence location ($P < 0.05$), with unmarried individuals and rural residents deriving a greater benefit from higher eGDR levels. Specifically, each IQR increase in eGDR was associated with a 66% reduction in the risk of MetS among rural residents (OR = 0.34 [95% CI: 0.29–0.42]) compared with a 41% reduction in urban residents (OR = 0.59 [95% CI: 0.46–0.75]). Similarly, each IQR increase in eGDR was associated with a 67% reduction in the risk of MetS among unmarried individuals (OR = 0.33 [95% CI: 0.21–0.53]) compared with a 57% reduction among married individuals (OR = 0.43 [95% CI: 0.36–0.50]).

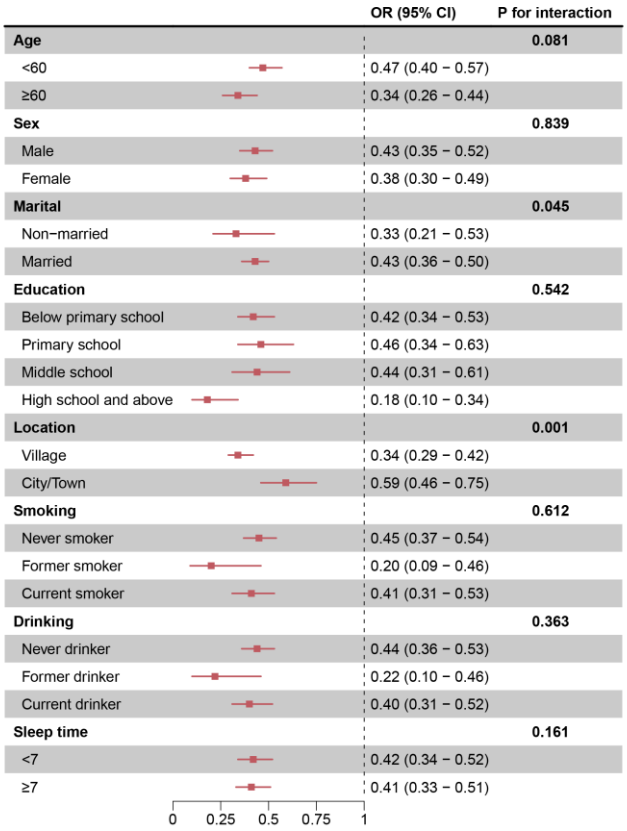

Fig. 3 The Association Between eGDR and MetS Varies by Subgroup.

Mediation Analysis

According to the mediation analysis results (Table A.2), eGDR had a significant indirect effect on MetS through BMI (estimated average causal mediated effect = −0.00252, $P < 0.001$), as well as a significant and negative direct effect (estimated average direct effect = −0.00547, $P < 0.001$). Approximately 29.08% of the total effect of eGDR on MetS was mediated by BMI (estimated proportion mediated = 0.29077, 95% CI: 0.19421–0.42000, $P < 0.001$).

ROC Curve Analysis

In this study, we evaluated the predictive performance of eGDR and its composite indicators (WC, HbA1c) for MetS (Table A.3 and Fig. 4). The area under the ROC curve (AUC) for eGDR in predicting MetS was 0.71 (95% CI: 0.69–0.73), which was higher than that for WC (AUC = 0.67, 95% CI: 0.65–0.69) and HbA1c (AUC = 0.52, 95% CI: 0.49–0.54). These findings suggest that eGDR, as a composite indicator, has a better predictive ability than any single MetS indicator.

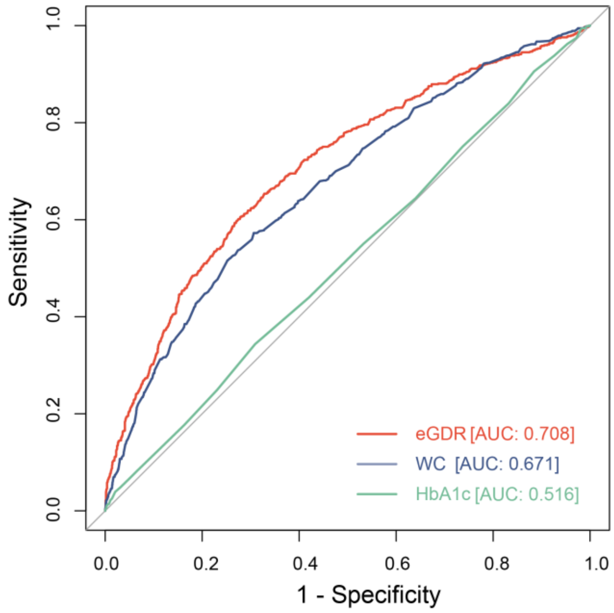

Fig. 4 Predictive Analysis Curves of eGDR, WC, and HbA1C for MetS.

Discussion

MetS is a complex cluster of metabolic disorders, and its prevalence is increasing steadily worldwide.(16) We observed a significant inverse association between eGDR and the risk of MetS. Additionally, marital status and residential location moderated this relationship, with unmarried individuals and rural residents benefiting more from higher eGDR levels. Furthermore, the dose–response analysis revealed a nonlinear negative relationship between eGDR and MetS risk. Higher eGDR levels were associated with a reduced risk of MetS up to a critical threshold of 11.88 (standard error = 0.17), beyond which the protective effect diminished.The ROC analysis further highlighted that eGDR has superior predictive power for MetS compared with single components such as WC and HbA1c.

Insulin resistance is one of the central pathological mechanisms underlying MetS.(17) Reduced insulin sensitivity leads to a diminished response to insulin, resulting in poor glycemic control. Prolonged insulin resistance not only disrupts glucose metabolism but also contributes to the development of abdominal obesity, hypertension, and dyslipidemia, key components that collectively define the criteria for diagnosing MetS.(18) Therefore, decreased insulin sensitivity (insulin resistance) significantly increases the risk of MetS. Studies show that hypertension, abdominal obesity, and elevated HbA1c levels are associated with reduced insulin sensitivity, which in turn increases the risk of developing MetS.(19-21) eGDR serves as a comprehensive marker for assessing insulin sensitivity and metabolic health by integrating WC, BP, and HbA1c levels. Our study identified eGDR as a factor that protected against MetS, consistent with previous findings. A five-year follow-up study conducted by Peng Juan et al. in 956 patients with type 2 diabetes found that an eGDR > 6.34 mg/kg/min served as a protective factor for renal outcomes.(22) Another study involving 104,697 patients with type 2 diabetes demonstrated that a higher eGDR (indicating lower insulin resistance) was associated with a lower risk of stroke and

mortality.(23) Furthermore, a six-year follow-up study indicated that a higher eGDR was linked to a reduced risk of cardiovascular disease, stroke, and cardiac events.(24) These findings reinforce our conclusion that eGDR levels are significantly associated with the risk of developing MetS.

Subgroup analyses indicated that married individuals and urban residents had a higher risk of MetS. For married individuals, changes in lifestyle, dietary habits, and increased social support following marriage can lead to greater energy and fat intake, which may increase the risk of obesity.(25-27) Additionally, the combined effects of aging and the added responsibilities of marriage can heighten the risk of hypertension, making married individuals more susceptible to MetS than their single counterparts.(28, 29) Urban residents are also at greater risk due to factors such as prolonged sedentary work, limited opportunities for physical activity, and higher consumption of processed foods, particularly those high in sodium and low in potassium.(30-32) These factors are closely associated with an increased risk of MetS.

Strengths and limitations

Our study has several strengths. First, it is the first prospective investigation of the relationship between eGDR and MetS risk. The findings further establish eGDR as a protective factor against MetS. Second, this study is based on a large, representative national sample, which enhances the generalizability and reliability of the results. Finally, the prospective cohort study design allows some elucidation of the causal relationship between eGDR and MetS, which provides valuable insights for reducing the incidence of MetS.

Nevertheless, the study has several limitations. First, it encompasses only a limited set of variables, potentially omitting other relevant factors. Second, despite adjusting for various confounders, the analysis could not account for genetic factors, dietary patterns, and other lifestyle influences due to data constraints. Third, the study was conducted solely among a Chinese population, which limits the generalizability of the findings to other countries and regions. Future research should involve additional prospective cohort studies to further elucidate the relationship between eGDR and MetS, thereby providing more comprehensive scientific evidence for MetS prevention.

Conclusion

In summary, our findings suggest that eGDR is significantly negatively associated with the risk of developing MetS. Improving insulin sensitivity may serve as effective strategies for the prevention of MetS. This research provides valuable insights for public health initiatives aimed at mitigating the risk of MetS.

Availability of data and materials

All data collected in CHARLS is maintained by the National School of Development at Peking University, Beijing, China. These data are accessible to researchers worldwide on the CHARLS project website (http://charls.ccer.edu.cn/charls/).

List of abbreviations

MetS : Metabolic Syndrome

eGDR : Estimated Glucose Disposal Rate

CHARLS : China Health and Retirement Longitudinal Study
WC : Waist Circumference
HbA1C : Glycated Hemoglobin A1C
TG : Triglyceride
BP : Elevated blood pressure
FPG : Elevated fasting plasma glucose
CRP : C-Reactive Protein
BMI : Body Mass Index
ROC: Receiver Operating Characteristic
AUC: Area Under the Curve
ADE : Average Direct Effect;
ACME : Average Causal Mediation Effect.

Acknowledgements:
We would like to express our sincere gratitude to Peking University for providing the CHARLS data and to all individuals involved in data collection and management.

Authors' contributions:

Lin Zhang: Conceptualization, Methodology, Data Curation, Writing - Original Draft.

Nian Cai: Formal Analysis, Investigation , Supervision.

Li Mo: Data Collection, Resources, Validation.

Xiaofang Tian: Software, Visualization, Formal Analysis.

Bohai Yu(Corresponding Author): Project Administration , Writing - Review & Editing

Funding:
This research did not receive any specific grant from funding agencies in the public, commercial, or not-for-profit sectors.

References

1. Roy R, Gautam AK, Singh NP, Kumar A. Metabolic Syndrome and Its Correlates Among Female Chronic Obstructive Pulmonary Disease Patients at a Rural Tertiary Health Care Center in Northern India. Cureus. 2022;14(8):e28611.
2. Fahed G, Aoun L, Bou Zerdan M, Allam S, Bou Zerdan M, Bouferraa Y, et al. Metabolic Syndrome: Updates on Pathophysiology and Management in 2021. Int J Mol Sci. 2022;23(2).
3. Saklayen MG. The Global Epidemic of the Metabolic Syndrome. Curr Hypertens Rep. 2018;20(2):12.
4. Mendrick DL, Diehl AM, Topor LS, Dietert RR, Will Y, La Merrill MA, et al. Metabolic Syndrome and Associated Diseases: From the Bench to the Clinic. Toxicol Sci. 2018;162(1):36-42.
5. Taverna MJ, Martínez-Larrad MT, Frechtel GD, Serrano-Ríos M. Lipid accumulation product: a powerful marker of metabolic syndrome in healthy population. Eur J Endocrinol. 2011;164(4):559-67.
6. Zieske AW, Tracy RP, McMahan CA, Herderick EE, Homma S, Malcom GT, et al. Elevated serum C-reactive protein levels and advanced atherosclerosis in youth. Arteriosclerosis, thrombosis, and vascular biology. 2005;25(6):1237-43.

7. Williams KV, Erbey JR, Becker D, Arslanian S, Orchard TJ. Can clinical factors estimate insulin resistance in type 1 diabetes? Diabetes. 2000;49(4):626-32.

8. Nyström T, Holzmann MJ, Eliasson B, Svensson AM, Sartipy U. Estimated glucose disposal rate predicts mortality in adults with type 1 diabetes. Diabetes Obes Metab. 2018;20(3):556-63.

9. Zabala A, Darsalia V, Lind M, Svensson AM, Franzén S, Eliasson B, et al. Estimated glucose disposal rate and risk of stroke and mortality in type 2 diabetes: a nationwide cohort study. Cardiovasc Diabetol. 2021;20(1):202.

10. Zhang Z, Zhao L, Lu Y, Xiao Y, Zhou X. Insulin resistance assessed by estimated glucose disposal rate and risk of incident cardiovascular diseases among individuals without diabetes: findings from a nationwide, population based, prospective cohort study. Cardiovasc Diabetol. 2024;23(1):194.

11. Linn W, Persson M, Rathsman B, Ludvigsson J, Lind M, Andersson Franko M, et al. Estimated glucose disposal rate is associated with retinopathy and kidney disease in young people with type 1 diabetes: a nationwide observational study. Cardiovasc Diabetol. 2023;22(1):61.

12. Zhao Y, Hu Y, Smith JP, Strauss J, Yang G. Cohort profile: the China Health and Retirement Longitudinal Study (CHARLS). Int J Epidemiol. 2014;43(1):61-8.

13. Zhao Y, Strauss J, Yang G, Giles J, Hu P, Hu Y, et al. China health and retirement longitudinal study–2011–2012 national baseline users' guide. Beijing: National School of Development, Peking University. 2013;2:1-56.

14. Helliwell R, Warnes H, Kietsiriroje N, Campbell M, Birch R, Pearson SM, et al. Body mass index, estimated glucose disposal rate and vascular complications in type 1 diabetes: beyond glycated haemoglobin. Diabetic Medicine. 2021;38(5):e14529.

15. Li Y, Gui J, Liu H, Guo LL, Li J, Lei Y, et al. Predicting metabolic syndrome by obesity- and lipid-related indices in mid-aged and elderly Chinese: a population-based cross-sectional study. Front Endocrinol (Lausanne). 2023;14:1201132.

16. Alberti KGM, Zimmet P, Shaw J. The metabolic syndrome—a new worldwide definition. The Lancet. 2005;366(9491):1059-62.

17. Ashok T, Puttam H, Tarnate VCA, Jhaveri S, Avanthika C, Trejo Treviño AG, et al. Role of Vitamin B12 and Folate in Metabolic Syndrome. Cureus. 2021;13(10):e18521.

18. Alexander CM, Landsman PB, Teutsch SM, Haffner SM. NCEP-defined metabolic syndrome, diabetes, and prevalence of coronary heart disease among NHANES III participants age 50 years and older. Diabetes. 2003;52(5):1210-4.

19. Grundy SM, Cleeman JI, Daniels SR, Donato KA, Eckel RH, Franklin BA, et al. Diagnosis and management of the metabolic syndrome: an American Heart Association/National Heart, Lung, and Blood Institute Scientific Statement. Circulation. 2005;112(17):2735-52.

20. Nakamura M, Yamazaki O, Shirai A, Horita S, Satoh N, Suzuki M, et al. Preserved Na/HCO3 cotransporter sensitivity to insulin may promote hypertension in metabolic syndrome. Kidney International. 2015;87(3):535-42.

21. Someya Y, Tamura Y, Kaga H, Sugimoto D, Kadowaki S, Suzuki R, et al. Insulin resistance and muscle weakness are synergistic risk factors for silent lacunar infarcts: the Bunkyo Health Study. Sci Rep. 2021;11(1):21093.

22. Peng J, Li A, Yin L, Yang Q, Pan J, Yi B. Estimated Glucose Disposal Rate Predicts Renal Progression in Type 2 Diabetes Mellitus: A Retrospective Cohort Study. J Endocr Soc. 2023;7(7):bvad069.

23. Zabala A, Darsalia V, Lind M, Svensson A-M, Franzén S, Eliasson B, et al. Estimated glucose disposal rate and risk of stroke and mortality in type 2 diabetes: a nationwide cohort study. Cardiovascular

diabetology. 2021;20:1-9.

24. Ren X, Jiang M, Han L, Zheng X. Estimated glucose disposal rate and risk of cardiovascular disease: evidence from the China Health and Retirement Longitudinal Study. BMC Geriatr. 2022;22(1):968.

25. Janghorbani M, Amini M, Rezvanian H, GOUYA MM, DELAVARI AR, Alikhani S, et al. Association of body mass index and abdominal obesity with marital status in adults. 2008.

26. Liu J, Garstka MA, Chai Z, Chen Y, Lipkova V, Cooper ME, et al. Marriage contributes to higher obesity risk in China: findings from the China Health and Nutrition Survey. Ann Transl Med. 2021;9(7):564.

27. Sobal J, Hanson KL, Frongillo EA. Gender, ethnicity, marital status, and body weight in the United States. Obesity (Silver Spring). 2009;17(12):2223-31.

28. Birditt KS, Newton NJ, Cranford JA, Ryan LH. Stress and Negative Relationship Quality among Older Couples: Implications for Blood Pressure. J Gerontol B Psychol Sci Soc Sci. 2016;71(5):775-85.

29. Liu H, Waite L. Bad marriage, broken heart? Age and gender differences in the link between marital quality and cardiovascular risks among older adults. J Health Soc Behav. 2014;55(4):403-23.

30. Najafipour H, Yousefzadeh G, Forood A, Karamouzian M, Shadkam M, Mirzazadeh A. Overweight and obesity prevalence and its predictors in a general population: A community-based study in Kerman, Iran (Kerman coronary artery diseases risk factors studies). ARYA Atheroscler. 2016;12(1):18-27.

31. Yang XL, Ouyang YF, Zhang XF, Su C, Bai J, Zhang B, et al. Waist Circumference of the Elderly over 65 Years Old in China Increased Gradually from 1993 to 2015: A Cohort Study. Biomed Environ Sci. 2022;35(7):604-12.

32. Yu D, Zhao L, Zhang J, Yang Z, Yang L, Huang J, et al. China Nutrition and Health Surveys (1982−2017). China CDC Weekly. 2021;3(9):193.

Click here to access/download
Supporting Information
Appendices fig.docx

Click here to access/download
Supporting Information
Appendices table.docx
